# Supplementary material for: Using Trait-Based Methods to Study the Response of Grassland to Fertilization in the Grassland in Semiarid Areas in the Loess Plateau of China
Source: Plants (Basel). 2022 Aug 4;11(15):2045. doi: 10.3390/plants11152045 (PMC9370584; doi:10.3390/plants11152045)
Supplement: Supplementary file 1 [file plants-11-02045-s001.zip › plants-1834213-supplementary.pdf]

Table S1 Relative biomass (%) of the six species under different N and P addition additions (mean ± s.e.; n = 3).

N	P	*B. ischaemum*	*L. davurica*	*S. bungeana*	*A. sacrorum*	*P. tanacetifolia*	*A. scoparia*
N0	P0	27.30±7.54	11.70±2.34	12.67±1.64	11.70±5.99	16.36±8.94	5.39±2.23
	P20	24.78±11.75	40.41±9.92	5.50±3.44	1.71±1.25	5.49±1.43	5.64±2.13
	P40	9.96±4.39	46.83±5.47	4.65±1.05	0.72±0.72	12.59±4.19	8.27±2.21
	P80	19.42±2.46	47.78±1.70	6.53±4.17	1.46±1.46	4.04±2.45	4.44±1.43
N25	P0	40.22±4.92	10.70±2.61	16.39±2.30	8.01±2.54	8.48±6.26	2.21±0.91
	P20	50.09±4.51	16.43±0.92	8.03±1.98	7.48±6.55	5.61±2.30	2.96±1.39
	P40	31.05±8.18	21.59±0.73	6.09±0.40	12.45±6.30	4.57±2.80	10.22±4.67
	P80	25.73±14.35	24.16±12.04	10.28±1.02	10.74±6.58	4.68±4.68	6.29±1.80
N50	P0	36.16±8.39	9.36±5.15	27.54±11.68	14.03±6.62	3.03±0.83	6.59±2.94
	P20	28.79±10.35	19.06±2.71	11.12±6.62	13.63±6.88	0.21±0.21	11.68±6.09
	P40	33.09±7.53	23.07±2.85	8.11±2.47	8.19±4.93	3.58±1.30	8.68±5.30
	P80	34.02±5.14	15.50±3.91	14.61±3.75	6.86±1.80	5.47±0.67	10.78±1.67
N100	P0	38.80±8.19	12.93±6.31	27.38±18.99	6.72±4.36	1.96±1.09	8.56±3.90
	P20	34.54±4.40	2.80±1.28	5.65±2.44	31.71±3.97	3.68±0.33	13.70±6.85
	P40	40.03±3.30	4.96±1.62	3.07±2.19	19.90±3.96	3.37±1.73	20.33±8.23
	P80	38.51±2.46	3.19±0.78	3.70±0.79	30.70±4.44	1.49±1.12	17.88±1.54
N		**(8.25)	***(9.43)	ns	**(10.28)	ns	*(7.23)
P		ns	**(7.23)	*(8.58)	ns	ns	*(5.64)
N × P		*(24.71)	*(14.70)	ns	**(14.08)	ns	ns

Note: Data in brackets are LSD values among different N and P addition treatments ($p \leq 0.05$). *, ** and *** indicate statistically significantly different at $p \leq 0.05$, $p \leq 0.01$, and $p \leq 0.001$, respectively. ns, means no significant difference. Same as following.

Table S2 Analysis of variance results (*F* values) for the effects of N addition (N), P addition (P), species and their interactions on leaf trait and maximum plant height (H_{max}). ns, *, ** and *** indicated non-significant, significant at $p < 0.05$, 0.01 and 0.001, respectively.

Factors	LN (g kg^{-1})	LP (g kg^{-1})	LN:P	SLA (cm^2 g^{-1})	LTD (g cm^{-3})	LDMC (g g^{-1})	H_{max} (cm)
N	16.50***	11.89***	27.81***	7.05***	12.18***	0.42ns	19.06***
P	1.15ns	80.17***	107.43***	4.28**	6.17***	1.19ns	1.82ns
Species	81.25***	59.97***	48.41***	85.48***	64.15***	23.37***	95.16***
N * P	2.05*	3.64***	1.50ns	0.13ns	1.33ns	0.57ns	2.41*
N * Species	2.08*	2.48**	2.78**	1.26ns	1.33ns	1.27ns	3.91***
P * Species	1.14ns	3.50ns	2.83**	0.58ns	2.50**	1.88*	1.01ns
N * P * Species	0.74ns	2.44***	1.07ns	0.50ns	1.12ns	0.74ns	0.88ns

Table S3 Community weighted leaf traits, maximum plant height (H_{max}) and community aboveground biomass under different N and P addition additions (mean ± s.e.; n = 3).

N	P	CWM_LN ($g\ kg^{-1}$)	CWM_LP ($g\ kg^{-1}$)	CWM_LN:P	CWM_SLA ($cm^2\ g^{-1}$)	CWM_LTD ($g\ cm^{-3}$)	CWM_LDMC ($g\ g^{-1}$)	CWM_H_{max} (cm)	Community AGB (g)
N0	P0	17.49±0.50	1.52±0.05	11.65±0.28	173.23±6.43	0.46±0.05	0.42±0.00	34.90±7.20	153.72±9.64
	P20	20.67±2.08	2.11±0.31	10.38±1.24	175.91±14.62	0.39±0.02	0.48±0.04	35.25±7.06	198.41±5.89
	P40	22.70±0.83	3.13±0.34	8.56±0.71	158.17±8.93	0.51±0.04	0.48±0.02	21.42±2.25	204.75±46.67
	P80	22.79±0.65	3.17±0.17	7.67±0.48	160.74±8.50	0.49±0.08	0.45±0.03	23.99±3.16	237.81±36.19
N25	P0	20.78±0.89	1.34±0.06	16.14±1.43	174.43±20.99	0.45±0.06	0.42±0.02	36.19±6.75	205.07±40.95
	P20	18.73±1.30	2.35±0.17	8.31±0.32	217.58±40.82	0.37±0.07	0.43±0.02	38.59±3.89	316.72±7.52
	P40	21.62±0.43	2.64±0.23	8.82±0.62	200.00±18.42	0.36±0.03	0.40±0.00	43.52±1.23	333.80±32.18
	P80	20.90±1.62	2.90±0.23	7.84±1.55	183.41±18.76	0.41±0.06	0.43±0.02	28.31±6.92	320.92±43.76
N50	P0	19.58±0.93	1.20±0.13	16.97±1.22	151.73±15.21	0.49±0.01	0.46±0.02	43.56±9.73	293.11±49.60
	P20	21.19±1.14	1.97±0.15	11.28±0.44	186.09±7.14	0.41±0.04	0.42±0.00	46.20±4.28	382.02±27.09
	P40	20.60±2.50	2.63±0.31	8.28±0.38	220.30±17.51	0.38±0.03	0.39±0.02	52.29±3.97	409.46±43.98
	P80	21.04±0.77	3.05±0.14	7.82±0.45	201.09±8.62	0.41±0.01	0.41±0.02	50.72±9.08	493.34±54.42
N100	P0	22.52±1.08	1.39±0.20	17.83±2.21	173.38±33.87	0.50±0.10	0.44±0.04	41.91±12.09	259.73±18.30
	P20	24.71±1.47	2.13±0.13	11.96±0.44	224.56±9.75	0.31±0.02	0.38±0.01	69.43±5.21	655.73±46.60
	P40	24.53±2.38	2.71±0.23	9.63±0.58	250.66±18.69	0.31±0.01	0.38±0.01	56.07±0.92	647.16±28.70
	P80	24.11±0.56	2.65±0.09	9.63±0.34	224.63±3.67	0.30±0.02	0.38±0.01	76.14±4.72	956.31±44.71
N		*(1.88)	ns	*(1.72)	ns	ns	ns	**(12.90)	***(91.1)
P		ns	***(0.32)	***(1.40)	**(20.46)	**(0.05)	ns	ns	***(66.4)
N × P		ns	ns	*(2.79)	ns	ns	ns	*(18.83)	***(137.3)
